# Clinicopathologic factors affecting discrepancies in HER2 overexpression between core needle biopsy and surgical biopsy in breast cancer patients according to neoadjuvant treatment or not

**DOI:** 10.7150/jca.59419

**Published:** 2021-06-04

**Authors:** Young-Hoon Jeong, Soon Auck Hong, Hye Shin Ahn, Soo kyung Ahn, Min Kyoon Kim

**Affiliations:** 1 Department of Surgery, Chung-Ang University College of Medicine, 224-1, Heukseok-dong, Dongjak-gu, Seoul 06973, Republic of Korea.; 2 Department of Pathology, Chung-Ang University College of Medicine, 224-1, Heukseok-dong, Dongjak-gu, Seoul 06973, Republic of Korea.; 3 Department of Radiology, Chung-Ang University College of Medicine, 224-1, Heukseok-dong, Dongjak-gu, Seoul 06973, Republic of Korea.; 4 Department of Surgery, Kangnam Sacred Heart Hospital, Hallym University College of Medicine, 1 Shingil-ro, Youngdeungpo-ku, Seoul.

**Keywords:** HER2, breast cancer, tumor heterogeneity, neoadjuvant treatment

## Abstract

**Background:** Accurate determination of human epidermal growth factor receptor 2 (HER2) status on breast core needle biopsy (CNB) tissue is important for determining neoadjuvant chemotherapies (NACs) for primary breast cancer. However, HER2 discrepancies occur before and after surgery, creating difficulties in the administration of appropriate NAC. This study aimed to identify the clinical factors affecting these discrepancies.

**Methods:** This study was conducted on patients with primary breast cancer who underwent breast surgery from January 2012 to December 2018 at the Chung-Ang University Hospital. HER2 status was analyzed using immunohistochemistry. HER2 was graded as 0 to +3, and all 2+ cases were evaluated for gene amplification. The patients were divided into two groups based on whether or not they received chemotherapy. Patient and clinical characteristics between the two groups were compared using the χ2 test and a logistic regression model.

**Results:** A total of 443 patients were evaluated; 384 patients (86.7%) did not receive NAC, and 59 patients (13.3%) received NAC. The HER2 discordance rate was 12.5% in the no NAC group and 23.7% in the NAC group. Most cases showed a change in HER2 status from negative in CNB to positive in surgical biopsy (SB). Clinicopathological factors affecting HER2 change in the no NAC group were larger tumor size and higher histologic grade. Meanwhile, poor response to prior chemotherapy affected HER2 change in NAC.

**Conclusion:** The overall accuracy of CNB in determining HER2 status was lower in the NAC group than in the no NAC group. Some clinicopathological factors may affect HER2 changes in each group at different levels. Based on the HER2 status at the time of diagnosis, the choice of HER2-targeted therapy may vary, even if this is not true. Future research on the effects of changes in HER2 status between CNB and SB on prognosis is needed.

## Introduction

Neoadjuvant chemotherapy (NAC) is one of the standard treatments for patients with primary breast cancer 1-3. With improved treatment results and access to neoadjuvant human epidermal growth factor receptor 2 (HER2)-targeted drugs, a precise preoperative assessment of tumor characteristics has become more important 4-6.

CNB is used to confirm HER2 status in the determination of preoperative treatments for primary breast cancer. The HER2/neu gene is amplified in 15-20% of breast cancers. HER2-targeting agents (e.g., trastuzumab, lapatinib, pertuzumab, trastuzumab emtansine (TDM-1)) are used to treat HER2-positive breast cancer and improve survival 7, 8. In the neoadjuvant setting, HER2-positive breast cancers with a non-pathologic complete response (non-pCR) have poorer outcomes than those with a pathologic complete response (pCR) 9. The correct determination of HER2 in CNB is crucial for improved patient outcomes.

Despite the overall high concordance rate of HER2 status between CNB and surgical biopsy (SB), several cases confuse surgical oncologists when choosing appropriate systemic therapy. If tumors with HER2-negative CNB results become HER2-positive after NAC, we lose the opportunity to improve patient prognosis by achieving a pCR with prior targeted therapy before surgery. In some other tumors, physicians have reported HER2-positive status on CNB but negative on SB. These readings may have different interpretations, depending on whether or not the patient received any systemic neoadjuvant treatment. We need to determine the prognostic impact and potential advantages of additional adjuvant therapy with trastuzumab on such patients. However, no study has analyzed HER2 discrepancies between CNB and SB according to prior chemotherapy.

This study examines the discrepancy between CNB and SB for HER2 status and analyzes the clinical factors indicating the differences *in situ*ations with or without preoperative chemotherapy.

## Methods

### Patients and Ethical Approval

We retrospectively reviewed the medical records of patients who underwent breast cancer surgery at the Chung-Ang University Hospital from January 2012 to December 2018. A pathology review was performed by a pathologist. We analyzed the clinicopathologic characteristics of patients such as age, body mass index (BMI), menopausal status, type of breast surgery, clinical T, N stage, histologic grade, ER, PgR, HER2 state, and HER2 discrepancies between CNB and SB.

**Figure 1:**
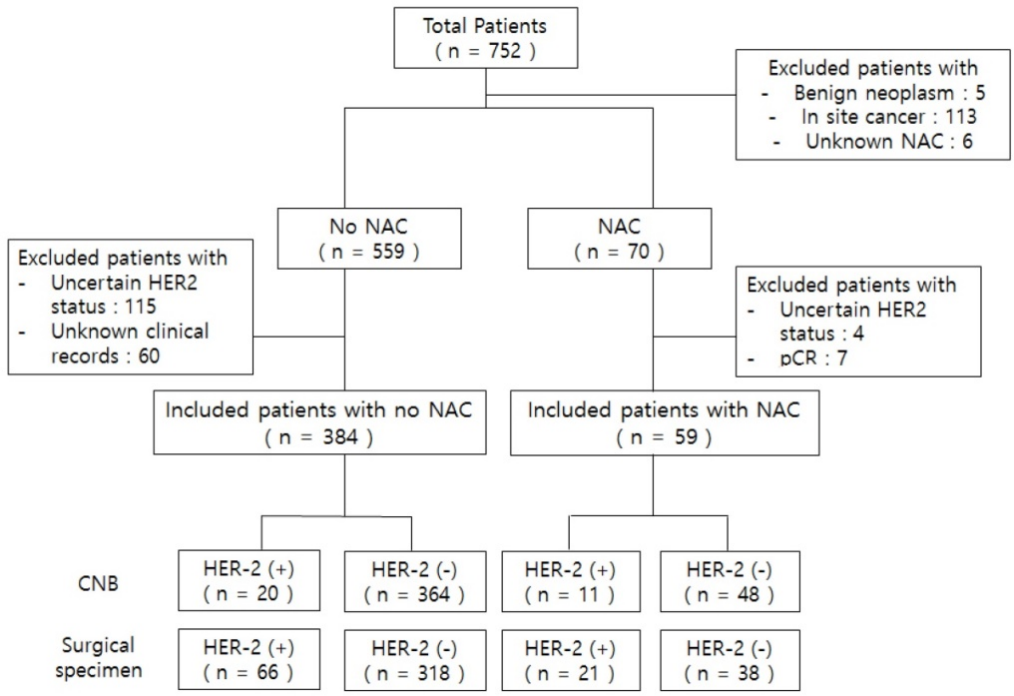
Flow chart of patients who participated in this study. *Uncertain HER2 status: IHC 2+ but no FISH or unknown HER2 status in CNB and surgical specimen. *Abbreviations: NAC, neoadjuvant chemotherapy; pCR, pathologic complete response after neoadjuvant chemotherapy.

We identified a total of 752 patients initially. We divided the total sample into two groups (with or without preoperative chemotherapy). We excluded patients with unknown NAC, *in situ* breast cancer, uncertain HER2 status, and those with pCR after NAC because residual tumor for the assessment of biomarkers was unavailable. Additionally, we excluded patients with unknown clinical factor records. A total of 443 patients met the inclusion criteria (Figure 1). This study was approved by the Institutional Review Board of the Chung-Ang University Hospital (IRB No. 2007-036-19326).

### Pathological Assessment

CNB specimens were retrieved from tumor centers by using an ultrasound-guided 14-gauge needle (Table 1). 348 patients (78.6%) were examined at Chung-Ang university hospital, and 95 patients (21.4%) were examined at another hospital. In 348 patients, 344 patients were examined CNB and 1 patient was examined vacuum assisted breast biopsy (VABB). Also, 3 patients were examined diagnostic surgical excision. In 95 patients, 87 patients were examined CNB, and 3 patients were examined VABB. Also 5 patients were examined diagnostic surgical excision. VABB used 11-gauge needle. Patients who underwent CNB obtained an average of 4.67 specimens. Patients who underwent VABB obtained an average 9.00 specimens. All immunostaining procedures and subsequent interpretations were performed at our institution. Pathology results were interpreted by a senior pathologist with abundant experience. IHC analysis was performed to evaluate the expression of ER, PgR, and HER2 in CNB and surgical specimens. ER and PgR expression was calculated as the percentage of cells showing a definite nuclear staining status. The cut-off value for ER and PgR positivity was ≥1% of tumor cells positive for nuclear staining.

HER2 status was also assessed in CNB and surgical specimens. HER2 status was scored as 0 to 3+ by IHC, according to the ASCO/CAP testing guidelines 10. For HER2, membranous staining was graded as negative (score 0 or 1+), weakly positive (score 2+), and strongly positive (score 3+). In weakly positive cases, HER2 gene amplification was performed. Cases were considered HER2-positive when the IHC score was 3+, or HER2 gene amplification was identified by fluorescence in site hybridization (FISH).

### Statistical analysis

The patient and clinical characteristics of the two groups were compared using the χ^2^ test. We calculated the accuracy of CNB compared to SB. We investigated whether there was a difference in HER2 discrepancy according to the presence of prior chemotherapy. Logistic regression models were used to analyze the patient and clinical characteristics affecting the change in HER2 status. P-values less than 0.05 were considered to indicate statistical significance. All statistical analyses were performed using SPSS version 20.0 software.

## Results

### Patient Characteristics According to NAC

A total of 443 patients were included in the study (Table 2). There were 59 patients (13.3%) with NAC and 384 patients (86.7%) without NAC; 80.5% of patients underwent breast-conserving surgery, and 19.5% underwent a total mastectomy. We performed axillary lymph node resection in 33.9% and sentinel lymph node biopsy in 66.1% of patients. The median age was younger in the NAC group (55.2±11.0 years in the no NAC group and 49.2±9.9 years in the NAC group). The BMIs between the two groups were similar (24.36 in the no NAC group and 24.56 in the NAC group). The majority of pathologic types were invasive ductal carcinoma (89.0%), followed by invasive lobular carcinoma (4.8%) and other subtypes (6.2%).

Mean tumor size was smaller in the no NAC group (20.2 mm in the no NAC group, 26.6 mm in the NAC group). There were 266 patients (60.0 %) in clinical T stages 0 or 1 and 177 patients (40.0%) in clinical stage 2 or higher. There were 274 patients (62.3%) in clinical N stage 0 and 167 patients (37.7%) in clinical N stage 1 or higher. ER positivity rate in SB was 293 (66.1%) in the no NAC group and 34 (7.7%) in the NAC group. The PgR positivity rate in SB was 274 (61.9%) in the no NAC group and 26 (5.9%) in the NAC group.

### Concordance of HER2 status between CNB and surgical specimens

The overall accuracy of CNB in determining HER2 status was lower in the NAC group than in the no NAC group (Table 3). In 384 patients who did not receive NAC, HER2 status was concordant between needle-biopsied and surgical specimens in 336 patients (87.5%). Forty-seven (12.2%) HER2-negative tumors changed to HER2-positive, while one (0.3%) HER2-positive tumor changed to HER2-negative. In 59 patients who received NAC, the HER2 concordance rate was 76.3%, and the HER2 discordance rate was higher than that for no NAC patients (23.7%). Twelve (20.3%) HER2-negative tumors changed to HER2-positive, while two (3.4%) HER2-positive tumors changed to HER2-negative. The HER2 concordance rate according to NAC showed a statistically significant difference (p-value=0.023). Most cases of HER2 discordance turned to HER2 overexpression in SB. The NAC group had eight patients using trastuzumab, which was positive for HER2 3+ in preoperative biopsies. Among them, there were two patients whose HER2 results were negative in surgical specimen. The other six still had positive HER2 results after surgery. However, more patients had no HER2-expression after NAC than the no NAC patients (p-value= 0.046). Since trastuzumab was used in the previous chemotherapy group, the rate of HER2 status change from positive to negative would have been higher.

### Clinicopathologic factors associated with HER2 change

**Figure 2:**
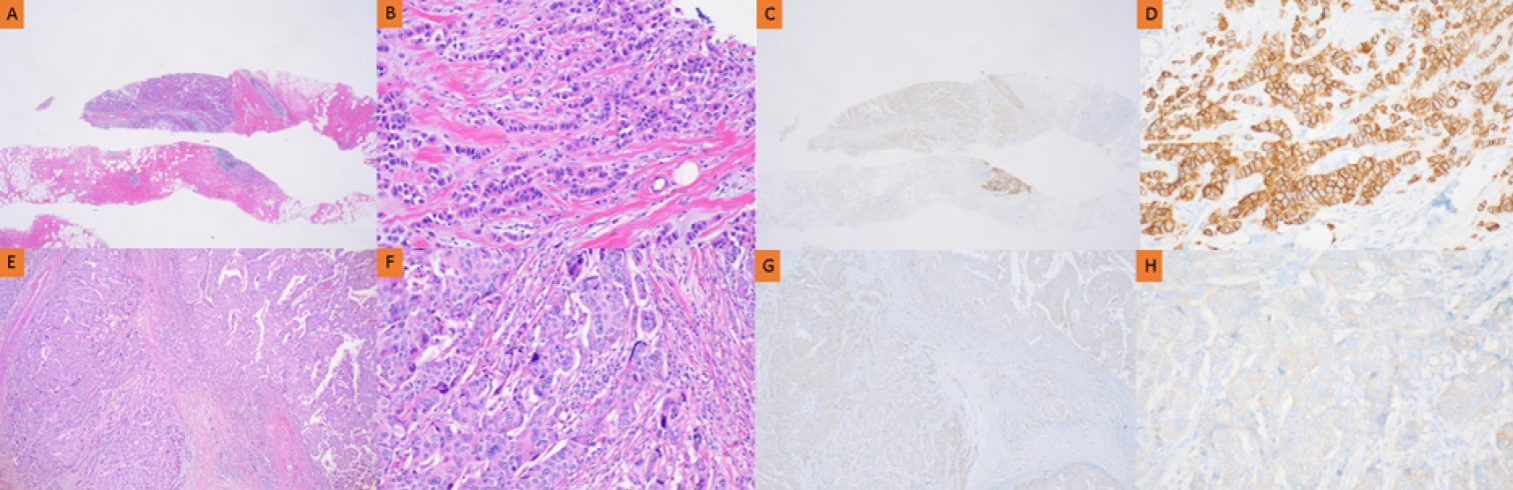
Pathology of HER2 discrepancy between CNB and SB in the no NAC group. Pathology of invasive ductal breast carcinoma in CNB (A, B, C, D) and SB (E, F, G, H). HER2 immunostaining showed strong complete membrane staining in >10% of tumor cells (C, D). However, HER2 immunostaining showed faint perceptible membrane staining in >10% of tumor cells (G, H).

Next, we analyzed the clinicopathological factors affecting HER2 discrepancies. In the no NAC group, menopausal status, tumor size over 2 cm, a higher histologic grade, and ER/PgR negativity were found to affect HER2 discrepancy in univariate analysis (Table 4). Multivariate analysis showed that tumor size (OR 2.518, p-value 0.012) and histological grade (OR-2.182, p-value 0.029) affected HER2 discrepancy (Table 4). Pathologic findings indicated that tumor heterogeneity according to tumor size and the histological grade was related to HER2 discrepancy (Figure 2).

In the NAC group, large residual tumors (yp T>1 cm) and the status of metastatic lymph nodes (yp N≥1) affected HER2 discrepancy (Table 4). However, multivariate analysis showed that no factor affected HER2 discrepancy in the NAC group (Table 5).

## Discussion

Several studies have evaluated the effects of HER2-targeted NAC. The NeoSphere trial 11 suggested that neoadjuvant pertuzumab is beneficial when combined with trastuzumab and docetaxel in progression- and disease-free survival at five years. The TRYPHAENA trial 12 showed that neoadjuvant pertuzumab and trastuzumab given concurrently or sequentially with an anthracycline-and carboplatin-based chemotherapy regimen resulted in an improved pCR rate. With these positive results, the National Health Insurance Service in Korea has started covering the cost of pertuzumab for neoadjuvant HER2-positive breast cancer patients. Furthermore, accurate determination of HER2 status before surgery has become crucial.

Reports show that the concordance rate (CR) of the biomarker status between CNB and surgical specimens is 79-100% 13. In a recent analysis, the CR of surgical specimens with CNBs for hormone receptor status had a 95.1% agreement. However, the CR of HER2 IHC was lower than that at 81.4% 14, similar to previous reports on HER2 discrepancy (ranging from 61-97.3%) 15. We treated the NAC or no NAC groups separately, and our results showed a higher rate (87.5%) of HER2 concordance in the no NAC group. The NAC group showed a higher rate (23.7% vs. 12.5%) of HER2 discordance.

The observed changes in HER2 status from positive to negative are rare. However, this phenomenon occurs more often in NAC cases and is observed more frequently with statistical significance, probably due to neoadjuvant HER2-targeted treatments. Possible underlying mechanisms include targeted therapies for chemo-sensitive tumor cells, which leave behind insensitive tumor cells with different biology in the residual tumor 16. Otherwise, changes in receptor expression of cells provide a survival mechanism for tumor cells, leading to cytotoxic drug resistance. Additionally, the expression of ER, PR, and HER2 are highly inter-dependent, and changing one receptor with NAC can modulate other receptors. Therefore, chemotherapy-induced hypoestrogenism may change HER2 status and estrogen and progesterone receptor expression 17.

Similar results were reported by a recent Japanese study showing that the rate of transition from HER2-positive to negative was three times greater after NAC 18. They evaluated the prognostic impact of changes in HER2 status in patients with HER2-positive tumors and found no difference in disease-free survival between patients with and without changes in HER2 status after NAC. Two other retrospective studies assessed the prognostic impact of changes in HER2 status after NAC in patients with primary breast cancer. Among patients with residual disease after NAC, Mittendorf et al. (25 patients) and Guarneri et al. (69 patients) demonstrated that patients with HER2 loss tended to have a higher risk of recurrence than those who maintained HER2 positivity 19, 20. Selective pressure on resistant HER2-negative clones and the induction of HER2 loss were the expected mechanisms of resistance and a reason for a poorer prognosis.

This study showed that the predictive factors affecting HER2 discrepancy in the no NAC group were related to menopause, breast surgery type, histologic grade, tumor size exceeding 2 cm, and ER/PgR negativity. Breast cancer is known as a heterogeneous disease with intertumoral and intra-tumoral heterogeneity. Cancer stem cells and their clonal evolution model could describe intra-tumor heterogeneity, and the tumor microenvironment could contribute to the complexity of these tumors. Structural pathological information on how well tubules form has been thought to lead to discordance between CNB and SB.

What we found in our study is that, rather than the change of HER2 to negative by treatment, the limitation of core biopsies, the tumor as a whole, and the heterogenicity of breast cancer itself, HER2 is often positively changed in surgical tissue. To reduce the discrepancy between CNB and surgical specimens, patients with the above-mentioned clinical factors need to undergo multiple CNB if possible. For accurate histologic diagnosis of breast cancer, at least four cores should be obtained using a 14-gauge needle 21.

Our study has some limitations. The case number was small, especially for NAC cases. Neoadjuvant HER2-targeted treatments were performed with trastuzumab only for almost all patients included in this study. In South Korea, pertuzumab has been covered by the National Health Insurance since 2019. Furthermore, we did not confirm the effects of HER2 status discrepancy between CNB and SB on patient prognosis. However, if a surgical oncologist experiences HER2 discrepancy between CNB and SB, our CNB's HER2 prediction tendency and clinically influential factors will help clinicians avoid confusion in deciding further treatment.

## Conclusion

The HER2 discordance rate was higher in the group with NAC (23.7%) than in the group without NAC (12.5%). Most HER2 discordance cases were HER2-negative in CNB but confirmed as HER2-positive in the surgical specimen. Clinicopathological factors affecting HER2 changes in the no NAC group were large tumor size (>2 cm) and high histologic grade. A large residual tumor (yp T>1 cm) and lymph node metastasis status (yp N≥1) had marginal effects on HER2 discrepancy in the NAC group. Future studies on the survival rate and prognosis according to these clinicopathological factors are necessary.

## Figures and Tables

**Table 1:** The method of preoperative breast cancer biopsy and average specimen number

	Biopsy method	Patient (N=443)	Specimen number
Our Hospital	14-Gauge Core needle biopsy	344 (77.7)	4.67
	11-Gauge Vacuum assisted biopsy	1 (0.2)	9.00
	Diagnostic surgical biopsy	3 (0.7)	
Another Hospital	14-Gauge Core needle biopsy	87 (19.6)	
	11-Gauge Vacuum assisted biopsy	3 (0.7)	
	Diagnostic surgical biopsy	5 (1.1)	

**Table 2:** Demographics of Included Patients (N=498)

	No NAC (N=384)	NAC (N=59)	P-value
**Age**			
Mean age, years	55.2	49.2	
Range	44.2-66.2	39.3-59.1	
**Body mass index**			
Mean, kg/m^2^	24.36	24.56	
Range	20.41-28.31	20.00-29.12	
**Menopause, n (%)**			
Pre-menopausal	156 (40.6)	33 (55.9)	0.027
Post-menopausal	228 (59.4)	26 (44.1)	
**Breast surgery type, n (%)**			
Total mastectomy	60 (15.6)	26 (44.1)	< 0.001
Breast conserving surgery	324 (84.4)	33 (55.9)	
**Axillary surgery type, n (%)**			
Axillary lymph node dissection	108 (28.1)	42 (71.2)	< 0.001
Sentinel lymph node biopsy	276 (71.9)	17 (28.8)	
**Histology, n (%)**			
Invasive ductal carcinoma (IDC)	341 (88.8)	53 (89.8)	0.910
Invasive lobular carcinoma (ILC)	18 (4.7)	3 (5.1)	
Others	25 (6.5)	3 (5.1)	
**Tumor size**			
Mean, mm	20.2	26.6	
Range	7.1-33.3	8.8-44.4	
**Clinical T stage, n (%)**			
T0, T1	237 (61.7)	29 (49.1)	0.103
T2, T3, T4	147 (38.3)	30 (50.9)	
**Clinical N stage, n (%)**			
N0	248 (64.6)	28 (47.5)	0.011
N+	136 (35.4)	31 (52.5)	
**Histologic grade, n (%)**			
1	70 (18.2)	6 (10.2)	< 0.001
2	207 (53.9)	11 (18.6)	
3	107 (27.9)	42 (71.2)	
**ER status, n (%)**			
Positive	293 (76.3)	34 (57.6)	0.002
Negative	91 (23.7)	25 (42.4)	
**PgR status, n (%)**			
Positive	274 (71.4)	26 (44.1)	< 0.001
Negative	110 (28.6)	33 (55.9)	
**HER2 status in core needle biopsy, n (%)**			
Positive	20 (5.2)	11 (18.6)	< 0.001
Negative	364 (94.8)	48 (81.4)	
**HER2 status in surgical specimen, n (%)**			
Positive	66 (17.2)	21 (35.6)	< 0.001
Negative	318 (82.8)	38 (64.4)	

**Table 3:** Accuracy of core needle biopsy compared to surgical specimens

		No NAC			NAC		
		N	%	Concordance rate	N	%	Concordance rate
cHER2(-)	sHER2(-)	317	82.6	87.5	36	61.0	76.3
cHER2(+)	sHER2(+)	19	4.9		9	15.3	
cHER2(+)	sHER2(-)	1	0.3		2	3.4	
cHER2(-)	sHER2(+)	47	12.2		12	20.3	
	Total	384	100		59	100	

**Table 4:** Predictive factors associated with HER2 discrepancy in the No NAC group

	Univariate			Multivariate		
	Concordance	Discordance	P-value	OR	95% CI	P-value
**Age**				1.502	0.469-4.817	0.494
≤40 yrs	30 (7.7)	2 (4.2)	0.237			
>40 yrs	361 (92.3)	46 (95.8)				
**Body mass index**						
<25 kg/m^2^	209 (62.2)	33 (68.8)	0.379			
≥25 kg/m^2^	127 (37.8)	15 (31.2)				
**Menopause, n (%)**				2.450	0.738-8.134	0.143
Pre-menopausal	143 (37.2)	13 (27.1)	0.041			
Post-menopausal	193 (50.3)	35 (72.9)				
**Histology, n (%)**						
Invasive ductal carcinoma (IDC)	297 (77.3)	44 (91.6)	0.761			
Invasive lobular carcinoma (ILC)	16 (4.2)	2 (4.2)				
Others	23 (6.0)	2 (4.2)				
**T stage, n (%)**				2.518	1.228-5.165	0.012
≤ 2 cm	217 (56.5)	20 (41.7)	0.002			
> 2 cm	119 (31.0)	28 (58.3)				
**N stage, n (%)**				0.411	0.193-0.874	0.021
N0	213 (55.5)	35 (72.9)	0.197			
N+	123 (32.0)	13 (27.1)				
**Histologic grade, n (%)**				2.182	1.083-4.393	0.029
Low (I-II)	253 (75.3)	24 (50.0)	<0.001			
High (III)	83 (24.7)	24 (50.0)				
**ER status, n (%)**				2.993	0.970-9.232	0.056
Positive	273 (71.1)	26 (54.2)	< 0.001			
Negative	63 (16.4)	22 (45.8)				
**PgR status, n (%)**				0.927	0.302-2.842	0.894
Positive	251 (65.4)	22 (45.8)	< 0.001			
Negative	85 (22.1)	26 (54.2)				

*Abbreviations: ALND, Axillary lymph node dissection; SLNB, Sentinel lymph node biopsy.

**Table 5:** Predictive factors associated with HER2 discrepancy in the NAC group

	Univariate			Multivariate		
	Concordance	Discordance	P-value	OR	95% CI	P-value
**Age**						
≤40 yrs	10 (22.2)	1 (7.1)	0.196			
>40 yrs	35 (77.8)	13 (92.9)				
**Body mass index**						
<25 kg/m^2^	30 (50.8)	8 (13.6)	0.516			
≥25 kg/m^2^	15 (25.4)	6 (10.2)				
**Menopause, n (%)**						
Pre-menopausal	27 (45.8)	6 (10.2)	0.259			
Post-menopausal	18 (30.5)	8 (13.6)				
**Histology, n (%)**						
Invasive ductal carcinoma (IDC)	39 (66.1)	14 (23.7)	0.354			
Invasive lobular carcinoma (ILC)	3 (5.1)	0 (0.0)				
Others	3 (5.1)	0 (0.0)				
**ypT stage, n (%)**				2.981	0.65-13.75	0.161
≤ 1 cm	8 (17.8)	6 (42.9)	0.051			
> 1 cm	37 (82.2)	8 (57.2)				
**ypN stage, n (%)**				1.720	0.37-7.93	0.487
N0	18 (30.5)	10 (16.9)	0.040			
N+	27 (45.8)	4 (6.8)				
**Histologic grade, n (%)**						
Low (I-II)	15 (33.3)	2 (14.3)	0.273			
High (III)	30 (66.7)	12 (85.7)				
**ER status, n (%)**						
Positive	24 (40.7)	9 (15.3)	0.471			
Negative	21 (35.6)	5 (8.5)				
**PgR status, n (%)**						
Positive	20 (33.9)	6 (10.2)	0.917			
Negative	25 (42.4)	8 (13.6)				
